# Biodegradable Magnesium Biomaterials—Road to the Clinic

**DOI:** 10.3390/bioengineering9030107

**Published:** 2022-03-05

**Authors:** Shukufe Amukarimi, Masoud Mozafari

**Affiliations:** Department of Tissue Engineering & Regenerative Medicine, Faculty of Advanced Technologies in Medicine, Iran University of Medical Sciences (IUMS), Tehran 1449614535, Iran; sh.amukarimi@gmail.com

**Keywords:** magnesium, biodegradability, biomaterials, degradation

## Abstract

In recent decades, we have witnessed radical changes in the use of permanent biomaterials. The intrinsic ability of magnesium (Mg) and its alloys to degrade without releasing toxic degradation products has led to a vast range of applications in the biomedical field, including cardiovascular stents, musculoskeletal, and orthopedic applications. With the use of biodegradable Mg biomaterials, patients would not suffer second surgery and surgical pain anymore. Be that as it may, the main drawbacks of these biomaterials are the high corrosion rate and unexpected degradation in physiological environments. Since biodegradable Mg-based implants are expected to show controllable degradation and match the requirements of specific applications, various techniques, such as designing a magnesium alloy and modifying the surface characteristics, are employed to tailor the degradation rate. In this paper, some fundamentals and particular aspects of magnesium degradation in physiological environments are summarized, and approaches to control the degradation behavior of Mg-based biomaterials are presented.

## 1. Introduction

It has been a long time since metallic biomaterials gained clinical significance [1]. Biomaterials are expected to be biocompatible in the human body’s internal environment containing aggressive ions. Some researchers, as a result, suggest using permanent metallic biomaterials, such as Ti-based alloys, CoCr alloys, and stainless steel [2,3,4,5]. These biomaterials are excellent choices for various medical applications, as they show high corrosion resistance, high strength [6], high hardness [7], and high fracture toughness [8]. On the other hand, the elastic modulus of most orthopedic implants made of these materials is greater than that of the natural bone, resulting in the stress-shielding phenomenon [9,10]. Several ions released from permanent biomaterials can also deteriorate biocompatibility. They may either be removed through a second surgery or remain in the human body; accordingly, several permanent biomaterials used in the market do not meet the requirements of the patient, leading to the development of degradable biomaterials [11].

Nowadays, degradable biomaterials play a crucial role in therapeutics, as they offer a steady resorption rate and, consequently, the best healing process. After providing adequate biomechanical support, resorbable biomaterials degrade gradually with no residues [12,13,14]. They fulfill the mission of promoting the healing process before being replaced by the host tissue [15,16]. No secondary operation is required, thereby eliminating the morbidity of the patient, extra costs, and the risk of new symptoms [17]. The reduction of mechanical support following the degradation process leads to transferring the loads from the orthopedic implants to the bones, thereby plummeting the risk of the reduction in bone density [18]. Even though bioresorbable polymers are candidate materials in tissue engineering and drug delivery, biodegradable metallic biomaterials offer an enhanced alternative for load-bearing applications [19,20]. Therefore, biodegradable metallic biomaterials are much more suited for use in load-bearing medical devices [21].

The most well-known biodegradable metals are iron (Fe), zinc (Zn), and magnesium (Mg), all of which are essential nutrients for human health [22,23]. The mechanical properties of Fe are the closest to that of a traditional permanent metallic implant, and its degradation rate is remarkably slow. Much as the degradation rate of Zn is moderate, the ductility and strength of this metal are low [24,25]. Studies following the implantation of Mg biomaterials indicate that the biocompatibility of Mg is desirable, and the degradation products of Mg can cause no disorder, inflammation, or allergic reactions to the human body [26,27,28,29,30]. However, the high corrosion rate, unexpected degradation, and structural failure of Mg-based biomaterials may trigger implant failure in some cases [31]. Numerous techniques, hence, have been utilized to alleviate such problems. The most important methods are adding non-toxic alloying elements to pure Mg and modifying the surface of these biomaterials [32,33,34,35]. By taking these methods into consideration, Mg-based biomaterials can be designed to degrade in a tailored behavior at different degradation rates to suit the requirements of a specific biomaterial for various applications [36,37,38]. This review article mainly focuses on the degradation behavior of Mg and its alloys for different biomedical applications.

## 2. Biodegradation Behavior of Magnesium-Based Materials

As a biodegradable material, magnesium oxidizes in contact with water on the grounds that the standard electrode potential of −2.372 V contributes to low corrosion resistance compared to other metals [39]. In the absence of water, an oxide film of MgO forms on the surface of Mg at room temperature (Equation (1)) [40]. Owing to this formed film, Mg indicates higher corrosion resistance in dry air. The thickness of this film is about 2.65 nm after one minute of exposure time to air [41]. Humidity can convert MgO film to Mg(OH)_2_ layer that is stable in pH values higher than 7 (Equation (2)) [42]. Both of these films on the surface of Mg are partly soluble in water; for this reason, they cannot protect the surface of Mg in acidic and neutral solutions. In contrast to MgO, Mg(OH)_2_, which is slightly soluble, precipitates on the surface of Mg and causes the alkaline pH shift of the solution.

(1) $$\mathrm{Mg}+\frac{1}{2}\;O_{2}\;\to \;\mathrm{MgO}\;$$

MgO + H_2_O → Mg(OH)_2_ (2)

Magnesium degradation in aqueous media begins with an anodic partial reaction: Mg loses two electrons to form Mg^2+^ Equation (3). As electrons are neither created nor destroyed in a chemical reaction, H_2_O gains these electrons to produce hydrogen gases and hydroxide ions Equation (4), resulting in the production of gas cavities and an increase in the pH of the solution in the surrounding tissues. Note that the overall reaction, Equation (5), yields one molecule of H_2_ for each atom of Mg dissolved. Finally, following this chemical reaction, a partially protective film forms on the surface of Mg, which limits the further migration of ions [43,44]. However, the production of hydrogen gases at the corrosion sites triggers the split of the deposited Mg(OH)_2_ precipitations from the surface and therefore prevents the formation of a uniform Mg(OH)_2_ film on the surface of Mg. The degradation of Mg is not, as a result, self-inhibited, and it continues until the complete degradation of the substrate [40,45,46].

Oxidation reaction: Mg → Mg^2+^ + 2e^−^ (3)

Reduction reaction: 2H_2_O + 2e^−^ → 2OH^−^ + H_2_ (4)

Product formation: Mg (s) + 2H_2_O (l) → Mg(OH)_2_ (s) + H_2_ (g) (5)

Based on the Pourbaix diagram for the Mg–H_2_O system at 25 °C, all domains of stability of Mg are below that of H_2_O; accordingly, Mg is a base metal and a reducing agent. This metal is significantly susceptible to corrosion in most inorganic acidic, neutral, and slightly alkaline solutions with a speed that decreases as the pH level increases [47]. In other words, Mg has a high affinity to react with H_2_O at different pH. At low pH levels, the corrosion potential matches the region where hydrogen is stable, resulting in the production of hydrogen gas [48]. At a pH level between 8.5 and 11.5, a protective layer of oxide or hydroxide forms on the surface of Mg. While in the presence of alkaline solutions, this metal is covered in an Mg(OH)_2_ layer, which protects it from fast corrosion. In fact, the corrosion resistance of magnesium and its alloys is closely linked to the passive layer [49].

As mentioned above, the formed magnesium hydroxide layer cannot preserve the surface of Mg from rapid corrosion, especially in an environment that contains a considerable amount of chloride ions. The reason for this is that Mg(OH)_2_ is converted into more soluble MgCl_2_, and the dissolution of Mg(OH)_2_ film accelerates the dissolution process [45,50]. The reactions can be expressed as below:

Mg + 2Cl^−^ → MgCl_2_ (6)

Mg(OH)_2_ + 2Cl^−^ → MgCl_2_ + 2OH^−^ (7)

It is noteworthy that, in a solution containing HCO_3_^−^ and HPO_4_^2−^, the corrosion products also consist of Mg/Ca carbonates and phosphates that might increase the precipitations on the surface of Mg, thereby decreasing the degradation rate of Mg-based materials. The reactions are presented as follows:

Mg^2+^ (or Ca^2+^) + OH^−^ + HCO_3_^−^ + (n − 1) H_2_O → Mg (or Ca) CO_3_·nH_2_O (8)

3Mg^2+^ (or Ca^2+^) + 2OH^−^ +2HPO_4_^2−^ + (n − 2) H_2_O → Mg_3_ (or Ca)(PO_4_)_2_·nH_2_O (9)

The distribution of degradation products of Mg is hardly uniform during the degradation process. Whereas Ca_3_(PO_4_)_2_ may appear preferentially, Mg_3_(PO_4_)_2_ may locate homogeneously at the corrosion sites. The main reason for this is that a large concentration of Mg ions avoids the nucleation of Ca_3_(PO_4_)_2_ [51,52]; it is, as a consequence, easier for Mg_3_(PO_4_)_2_ to precipitate all over the surface. Following the coverage of the Mg surface with a protective layer of Mg_3_(PO_4_)_2_, the nucleation of Ca_3_(PO_4_)_2_ occurs, and a non-uniform distribution of Ca_3_(PO_4_)_2_ forms at the product layer [51]. Finally, the complete degradation of Mg is caused by the equilibrium between the production and dissolution of degradation products, besides the conversion of the active layer into a passive one [25].

## 3. Mg Corrosion in Simulated Body Environments

One of the most important factors in evaluating the degradation behavior of magnesium-based biomaterials is finding a suitable physiological fluid, as the degradation rate of these biomaterials differs significantly in various types of simulated body fluids. To simulate a human body environment, different media, notably physiological saline (0.9% NaCl) solution, Ringer’s solution (RS), phosphate-buffered saline (PBS), simulated body fluid (SBF), Hank’s balanced salt solution (HBSS), Earle’s balanced salt solution (EBSS), and Dulbecco’s Modified Eagle medium (DMEM), are widely used. Each simulated body solution contains a specific amount of components that could trigger the formation of different degradation products, pathways, and mechanisms [53]. By way of illustration, the degradation product layer formed on the surface of Mg exposed to Ringer’s solution mainly consists of magnesium calcite and brucite, as opposed to the layer formed on the surface of Mg immersed in Hank’s solution, which included calcium phosphate, calcite, and brucite [54]. However, an XPS investigation carried out on the surface of Mg revealed that the same components, including MgO, Mg(OH)_2_, and MgCO_3_, were formed after exposure to SBF, HBSS, and DMEM [55].

By and large, a suitable simulated body solution ought to consist of three main parts: inorganic salts, buffering systems, and organic elements. To measure the degradation behavior of Mg and its alloys, physiological saline (0.9% NaCl) solution was used in several studies, most of which showed a striking difference between in vitro and in vivo results [56], compared to SBF and HBSS that indicated more reliable results [57]. RS is a solution with at least three different recipes: with lactate, with HCO_3_^−^, and without HCO_3_^−^ [58]. The composition of this solution is not well-defined for corrosion testing of metallic implants [59], resulting in substantially different corrosion resistance. In most cases, the corrosion rate would be high due to the insufficient inorganic ions in Ringer’s solution, as opposed to interstitial and human body fluids. In the case of magnesium, the corrosion rate would decelerate owing to the combination of HCO_3_^−^, Ca^2+^, and alkaline pH at the Mg interface, which forms CaCO_3_ [60].

Despite the fact that PBS has been extensively used as the corrosion testing medium of Mg and its alloys [61,62,63,64], it is not generally a suitable solution to simulate or predict the in vivo degradation behavior of Mg, since phosphate with Mg^2+^ can create insoluble precipitation on the surface of the metal, which can produce inaccurate results [53,65]. Mena-Morcillo et al. [66] investigated the degradation of AZ31 and AZ91 Mg alloys in SBF, Hanks’, and Ringer’s solutions. They found out that the corrosion products precipitated on the surface of Mg alloys in Hanks’ media showed higher stability compared to SBF and Ringer’s solutions; as a result, those Mg alloys exposed to Hanks’ media were less affected. SBF, HBSS, and EBSS mainly include similar inorganic components with slightly different concentrations [67]. Although SBF has been used to test the apatite-forming ability of biomaterials [68,69], the absence of organic compounds makes it difficult to obtain accurate results, in that the degradation performance of Mg and its alloys is considerably different under the cell culture environment [70,71]. Moreover, in different studies in which the corrosion rate of pure Mg was assessed in SBF, radically different results were obtained [48,72,73,74,75], reducing the popularity of this solution for corrosion testing. HBSS is reported to be simple compared to DMEM, which contains organic components [76]. In a recent study, pure Mg was exposed to SBF, HBSS, and DMEM under cell culture conditions with CO_2_ gassing. The results indicated that SBF- and DMEM- based media indicated a higher buffering capacity than HBSS, and the degradation rate of Mg was highest in HBSS [76]. In another research study, the corrosion rate of pure Mg exposed to HBSS was very high [77].

EBSS has been used widely for in vitro testing of Mg and its alloys [78,79,80,81]. It is believed that the degradation rate of Mg biomaterials in EBSS is comparable to in vivo conditions [82,83,84,85,86]. Walker et al. [87] immersed pure Mg and five Mg alloys in EBSS, MEM, and MEM-containing BSA (MEMp) and implanted the samples in Lewis rats. After 21 days, the results indicated that the corrosion rate of samples immersed in EBSS buffered with sodium bicarbonate was similar to that obtained in vivo. In addition to EBSS, cell culture media, such as DMEM and MEM, are preferable to investigate the corrosion behavior of Mg-based biomaterials [88,89,90].

Another crucial factor in simulated body solutions is the buffering system. A natural buffer system, which consists of plasma protein buffers, HPO_4_^2−^, and HCO_3_^−^, controls the pH level in the human body [91,92]; by the same token, an appropriate buffering system can control the pH of a buffer solution. NaHCO_3_/CO_2_ buffer, 4-(2-hydroxyethyl)-1-piperazineethanesulfonic acid (HEPES), and Tris-HCl (Tris Hydrochloride) are the most frequently used buffers for in vitro studies of Mg [37,84,93]. HEPES buffer increases Mg corrosion by a factor of up to four times compared to NaHCO_3_ buffering alone in DMEM, EBSS, and simple salt solutions under the same conditions [94]. HEPES in testing solutions affects the nucleation process and reduces the formation of carbonate and phosphate in the degradation layer; in this way, the protective layer on Mg is destabilized, a less dense degradation layer is produced, and the progressive diffusion of aggressive ions is allowed [95,96]. Besides that, HEPES is reported to be a selective dissolution of Ca-containing phases on glass-ceramics. When pure Mg is exposed to Tris-HCl Buffer in SBF, it is more sensitive to pitting corrosion. For one thing, Tris-HCl prevents the formation of corrosion products on the surface of Mg alloy. For another thing, Tris increases the degradation rate of pure Mg by a factor of ten during earlier stage exposure. The presence of Tris-HCl buffer in simulated body fluid makes pure Mg extremely susceptible to pitting corrosion [93].

Unlike Tris and HEPES, the HCO_3_^−^/CO_2_ buffering system is preferred for in vitro assays on the grounds of the similarity to the regulation of the pH of the body. CO_2_ in the testing system not only promotes the formation of carbonate on the surface of Mg but also triggers a stable pH through the equilibrium of HCO_3_^−^/CO_2_. A carbonated film formed in the presence of CO_2_ under aqueous conditions is thicker than an Mg(OH)_2_ film formed in the absence of CO_2_, thereby decelerating the degradation rate [53]. Törne et al. [97] compared the effect of HEPES and HCO_3_^−^/CO_2_ on the degradation of Mg. They found out that m-SBF(HEPES) increased the corrosion rate of Mg, whereas the corrosion mechanism of Mg in m-SBF(CO_2_) was similar to in vivo corrosion mechanism.

A number of cell culture media with small molecule organic compounds and proteins have been designed to evaluate the corrosion behavior of Mg. With the appearance of these compounds in the solutions, the complexity of corrosive media increases because the corrosive media resembles the real body fluid more closely. The corrosion resistance of Mg, in most cases, could increase [60]. Yan et al. [98] evaluated the synergistic effects of protein and glucose on the degradation of Mg. They reported that the degradation of Mg was inhibited significantly, as the synergistic effect of protein and glucose limited the adsorption of aggressive Cl^−^ to a certain extent.

An investigation assessed the stress-corrosion-cracking susceptibility of Mg–1Zn alloy in PBS, bovine calf serum (BCS), modified simulated body fluid (m-SBF), and DMEM as a case in point [99]. It was reported that those samples immersed in PBS showed serious pitting corrosion, whereas those samples exposed to BCS and DMEM indicated higher resistance to corrosion. In another research, Mei et al. studied the corrosion of Mg exposed to albumin-containing HBSS. It was demonstrated that the presence of BSA resulted in rapid corrosion of pure Mg as the formation of the protective film on the surface of corroded Mg decelerated during the first hours of immersion [90]. One of the reasons behind these results may be the influence of organic compounds on the degradation product layer. Hou et al. [100] chose fetal bovine serum (FBS), L-alanyl-L-glutamine (L-Ala-LGln), L-glutamine (L-Gln), and L-ascorbic acid (L-AA) to illustrate the influence of organic molecules on the degradation behavior of pure magnesium under cell culture conditions. It was found that organic components have a major influence on the formation of the degradation layer. In the “inner” layer, the addition of organic components promoted the formation of phosphate (Mg–PO_4_ and Ca–P salts) during immersion; conversely, in the “outer” layer, these components assisted the precipitation of nesquehonite rather than hydromagnesite. However, the effects of many other organic compounds and proteins on the degradation behavior of Mg have yet to be explored.

## 4. Current Status of Mg-Based Biomaterials

Biomaterials, ideally, ought to degrade following tissue healing, and, furthermore, the biodegradation process should have no adverse effects on human health. Magnesium as a biodegradable material can play an important role in the biomedical field. Be that as it may, the degradation of untreated Mg in the physiological environment would indicate a high degradation rate, hydrogen evolution, and an increase in the pH of local tissues, which could harm surrounding tissues [101,102,103,104]. Accordingly, Mg resorption must be controlled, normally, by introducing particular alloying elements to magnesium and modifying the surface of biomaterials. Using these techniques, modified Mg-based devices can be utilized for cardiovascular [105,106,107,108], musculoskeletal, and orthopedic applications [109,110,111]. It can also be used in other oral and general applications [112].

### 4.1. Selection of Alloying Elements for Controlling the Degradation Behavior

The addition of alloying elements has a direct influence on the degradation behavior of Mg biomaterials. A case in point is the degradation rate of ZJ41 Mg alloy, which is very fast compared to AZ31 Mg alloy [113]. By and large, the design of Mg-based biomaterials needs meticulous care. For one thing, alloying elements might react with magnesium and create intermetallic phases, which dissolve in the Mg matrix or distribute along the grain boundaries, leading to different microstructures and degradation rates [114]. For another thing, the metallic ions released from Mg alloys must be biocompatible. Considering these two factors, we deem that the most popular alloying elements for Mg are calcium (Ca), zinc (Zn), manganese (Mn), strontium (Sr), lithium (Li), zirconium (Zr), yttrium (Y), and aluminum (Al). The effect of these alloying elements on the degradation of Mg is summarized in Table 1.

Ca is the main part of human bones and is vital for the life of human beings [128]. Ca is mainly found in bones and teeth [129,130,131]. The release of calcium ions regulates the activation of osteoclasts and osteoblasts, thereby facilitating bone regeneration in vitro and in vivo [132,133]. The addition of this element to magnesium alloys can enhance the corrosion resistance, mechanical properties, microstructure, and electrochemical behavior of Mg–Ca alloys [134,135,136]. Ca has an impact on the development of texture during rolling or extrusion, causing weaker textures without a strong alignment of basal planes. Such textures are known to show lower anisotropic mechanical behavior and also higher ductility [137]. The in vitro and in vivo degradation behavior of binary Mg–xCa alloy (x = 0.5 or 5.0 wt.%) was determined by Makkar et al. [116]. The in vitro study showed that the degradation rate differed linearly, with the Ca content indicating higher degradation, increased pH, and more hydrogen gas evolution in Mg–5.0Ca alloy. Moreover, in vivo studies revealed rapid degradation, prolonged inflammation, and higher initial corrosion rate in Mg–5.0Ca compared to Mg–0.5Ca alloy.

Zinc is an essential trace element that people need to stay healthy. This element can help in the normal functions of many enzymes, the normal growth of gonads, the treatment of bacterial infections, the improvement of cognitive abilities, neurotransmission, and synapse formation [138,139,140]. Studies have indicated that Mg–Zn alloys possess great mechanical properties, biocompatibility, and higher corrosion resistance [141]. Apart from that, the addition of Zn to Mg alloys can significantly reduce H_2_ evolution [142,143]. However, depending on the Zn content in binary Mg–Zn alloys and the phase distribution, the corrosion resistance of Mg–Zn alloys extensively differs. Zhang et al. [144] implanted Mg–6Zn alloy rods in the body of rabbits. The results indicated that the Mg alloy could be gradually absorbed in vivo at the degradation rate of 2.32 mm/yr, obtained by the weight-loss technique, with no disorders of the heart, liver, kidney, and spleen. Also, six weeks after implantation, subcutaneous H_2_ gas accumulated by the degradation of the alloys disappeared without discernable adverse influences.

In the human body, Mn is required for the normal functionality of the brain, nervous system, enzyme, and cellular homeostasis [145,146,147]. In Mg alloy implants, Mn plays the role of enhancing the corrosion resistance of the alloys without deteriorating mechanical integrity [148] Yu et al. [149] investigated the texture, microstructure, and mechanical properties of Mg–3Mn alloys. It was indicated that the samples showed weakened basal texture, refined microstructure, good yield strength, and high tensile elongation.

Strontium is considered one of the potential candidates for orthopedic applications in that this element can promote the growth of osteoblast cells [150,151,152]. A certain amount of Sr in Mg alloys can enhance the corrosion resistance [153] and mechanical strength of the alloys [154]. Jiang et al. [155] examined the degradation performance and biocompatibility of four binary MgSr alloys (Mg–xSr, x = 0.2, 0.5, 1, and 2 wt.%), together with four ternary MgCaSr alloys (Mg–1Ca–xSr, x = 0.2, 0.5, 1, and 2 wt.%) through direct culture with bone-marrow-derived mesenchymal stem cells (BMSCs). It was indicated that Mg–1Sr and Mg–2Sr alloys showed the lowest degradation rates in comparison with the other binary MgSr and ternary MgCaSr alloys. Ternary MgCaSr alloys revealed an enhanced BMSC adhesion on their surfaces in comparison with binary MgSr alloys, except for Mg–1Ca–0.2Sr alloy. Furthermore, Mg–1Sr, Mg–1Ca–0.5Sr, and Mg–1Ca–1Sr alloys presented the best performance concerning the degradation and BMSC performances between the above mentioned alloys.

**Figure 1:**
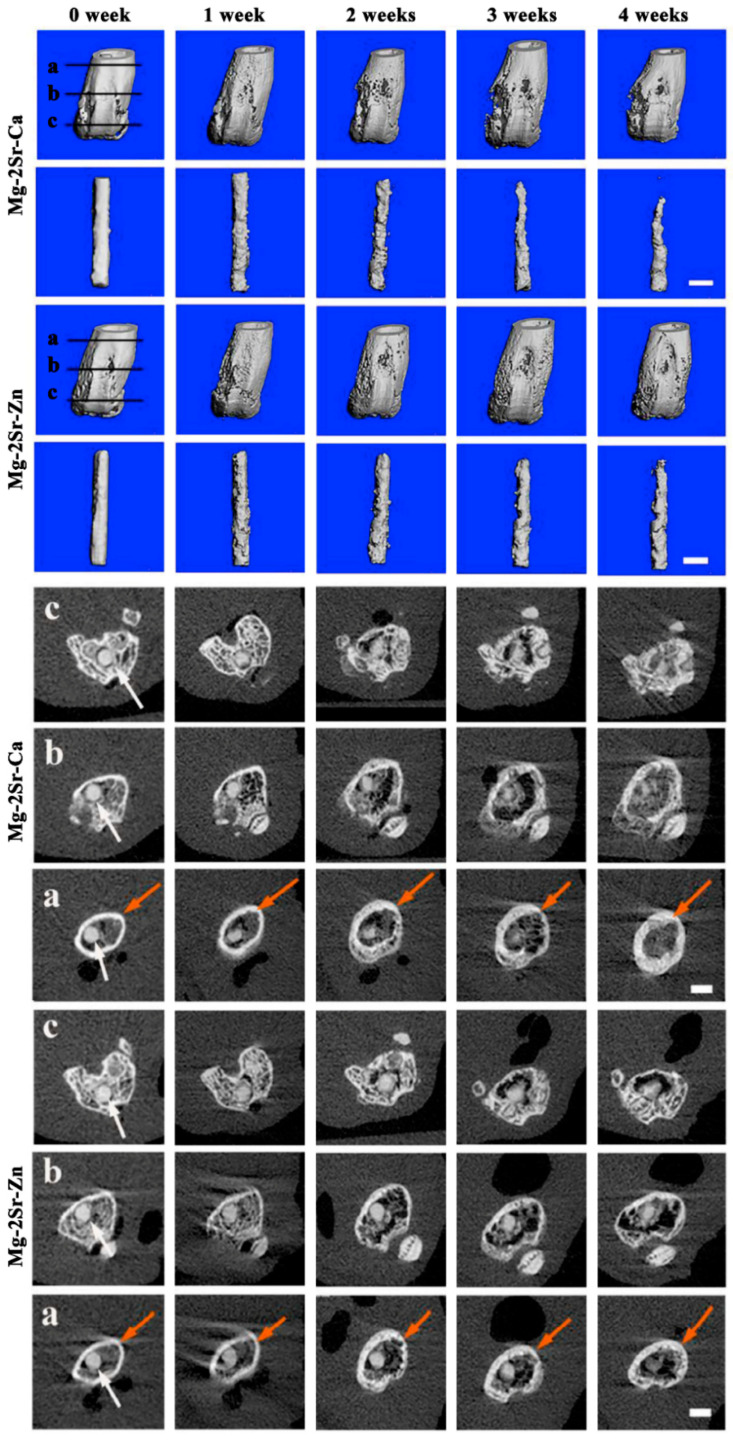
Three-dimensional reconstruction of the femora of mice, intramedullary Mg–2Sr–Zn and Mg–2Sr–Ca pins and two-dimensional cross-sectional images of the femora of mice in various places after surgery, corresponding to the straight black lines with embedded Mg–2Sr–Zn and Mg–2Sr–Ca pins (white arrows) at various post-operation time points. (**a**) The proximal part of the distal femur, (**b**) middle part of the distal femur, and (**c**) distal part of the distal femur. The bar length is 1.0 mm. As is indicated, localized degradation of the bio-materials at the surface of the rod can be seen in both trabecular and cortical bone regions one week after implantation. In the bone-marrow-cavity area, more rapid degradation was found in comparison with the distal areas, and the in vivo degradation of Mg–2Sr–Ca alloy rods was faster than that of Mg–2Sr–Zn alloy rods. Reprinted with permission from Ref. [156]. Copyright 2020, KeAi. [156].

Chen et al. [156] prepared Mg–2Sr–Zn and Mg–2Sr–Ca alloys and then investigated their degradation behavior. In this study the addition of Zn and Ca improved the in vitro and in vivo corrosion resistance compared to the binary Mg–2Sr alloys. While the in vivo corrosion rates for Mg–2Sr–Zn and for Mg–2Sr–Ca were 0.85 mm/year and 1.10 mm/year, this one for Mg–2Sr was 1.37 mm/year. The degradation of these rods via the three-dimensional reconstruction of the femora with implants and two-dimensional cross-sectional micro-CT images is shown in Figure 1. As is demonstrated, one week after implantation, localized degradation of the biomaterials at the surface of the rod can be seen in both trabecular and cortical bone areas. In the bone-marrow-cavity region, more rapid degradation occurred compared with the distal regions. Moreover, the in vivo degradation of rods made of Mg–2Sr–Ca alloy was faster than that of Mg–2Sr–Zn alloy rods.

Although lithium is not officially considered a micronutrient [157], it is remarkably effective against a wide spectrum of bacteria and has potent immune-stimulating capabilities [158]. It is said that lithium can be utilized as a promising bioactive element so as to promote the osteogenesis process because Li-based scaffolds could improve bone regeneration and stimulate bone-marrow mesenchymal stem cells’ osteogenesis [159]. This element is used as augmentation therapy for depression and as a typical mood stabilizer for the treatment of bipolar disorder [160]. While low Li could reduce life expectancy, cause problems in behavior, impair the reproductive function of the organism, and slow down the growth of the cells, high doses might trigger intoxication and result in pathological functional changes of individual organs or body systems [161]. The addition of Li in Mg alloys facilitates the activation of the prismatic slips and enhances the microstructures of Mg–Li alloys [36,162]. The most prominent properties of the Mg–Li alloys are their superior ductility and formability, which make them a great candidate for cardiovascular stent applications. Zhou et al. [163] studied Mg–3.5Li and Mg–8.5Li binary alloys to evaluate their degradation behavior for cardiovascular stent application. However, the strength of Mg–Li binary alloys was not adequate, owing to the presence of Li. Accordingly, Al and REEs were added to produce Mg–Li–Al ternary and Mg–Li–Al–RE quarternary alloys. The results of cytotoxicity tests revealed that the Mg–3.5Li–2Al–2RE, Mg–3.5Li–4Al–2RE, and Mg–8.5Li–2Al–2RE alloys suppressed vascular smooth-muscle cell proliferation five days post-incubation, whereas the Mg–3.5Li, Mg–8.5Li, and Mg–8.5Li–1Al alloys did not cause any problems. The Mg–Li-based alloys in the case of human umbilical vein endothelial cells indicated no considerable reduction in cell viabilities except for the Mg–8.5Li–2Al–2RE alloy, with no clear contrasts in cell viability between various culture periods.

In a number of studies, it has been shown that Zr presents desirable osteocompatibility, biocompatibility, corrosion resistance, and low ionic cytotoxicity [164,165,166]. The addition of Zr into Mg alloys can effectively refine the Mg grain size [164]. Mg alloys containing Zr often show good damping properties, lower hot-cracking tendency, corrosion resistance, and mechanical property [167]. Sayari et al. [168] investigated the effect of 0.7 wt.% Zr addition on the superplastic behavior and microstructure of extruded Mg. They found that the Mg–0.7Zr alloy indicated superplastic behavior after moderate deformation imposed by the extrusion process for all improved strength. They also reported that a bimodal microstructure was developed and the grain size was decreased due to the addition of Zr.

Extensive use of REEs is reported to impact human health [169]; however, several studies have shown the antibacterial and antifungal activities of these elements [170,171]. In Mg alloys, REEs have indicated great potential in improving formability, enhancing ductility, weakening sharp basal textures, and refining grains [172]. REEs also could improve the corrosion resistance of Mg alloys, as a stable corrosion product layer could be formed on the surface of Mg [173]. Azzeddine et al. [174] studied the corrosion behavior of Mg–1.43La, Mg–1.44Nd, Mg–0.63Gd, Mg–0.41Dy, and Mg–0.3Ce (wt.%) alloys. It was shown that the corrosion resistance of the alloys was decreasing in the following order: Mg–0.41Dy, Mg–0.63Gd, Mg–0.3Ce, Mg–1.44Nd, and Mg–1.43La. It is reported that the presence of a high fraction of the Mg_12_La phase acted as an anodic phase along the grain boundaries in the Mg–1.43La alloy and triggered severe pitting corrosion, while the formation of the Dy_2_O_3_ oxide inhibited the Mg–0.41Dy alloy from pitting corrosion and led to high corrosion resistance. In another study, Liu et al. [125] individually added sixteen types of REEs into pure Mg to compare the impact of each type of REEs on the corrosion behavior, mechanical property, microstructure, and biocompatibility of Mg materials. The results indicated that the addition of various REEs with suitable concentrations into Mg could enhance the general behavior of Mg from several aspects. The corrosion resistance of Mg–light REE alloys was enhanced compared to Mg–heavy REE alloys. The mechanical properties of Mg–RE binary alloys were significantly adjusted, and Mg–RE sample alloys indicated no cytotoxic influence on MC3T3-E1 cells.

While Al was believed to be nontoxic, recent studies indicate that this metal can negatively affect human health, such as brain diseases (multiple sclerosis, Parkinson’s disease, and Alzheimer’s disease) [175,176,177]. Moreover, it could disrupt the pro-oxidant/antioxidant balance in tissues resulting in physiological and biochemical dysfunctions on the grounds of an excessive reactive oxygen species generation [178]. Al, however, has the most favorable influence on Mg alloys. It can enhance corrosion resistance, fatigue strength, castability, and hardness [179,180,181,182].

### 4.2. Surface Treatment for Controlling the Biodegradation Behavior of Mg and Its Alloys

**Figure 2:**
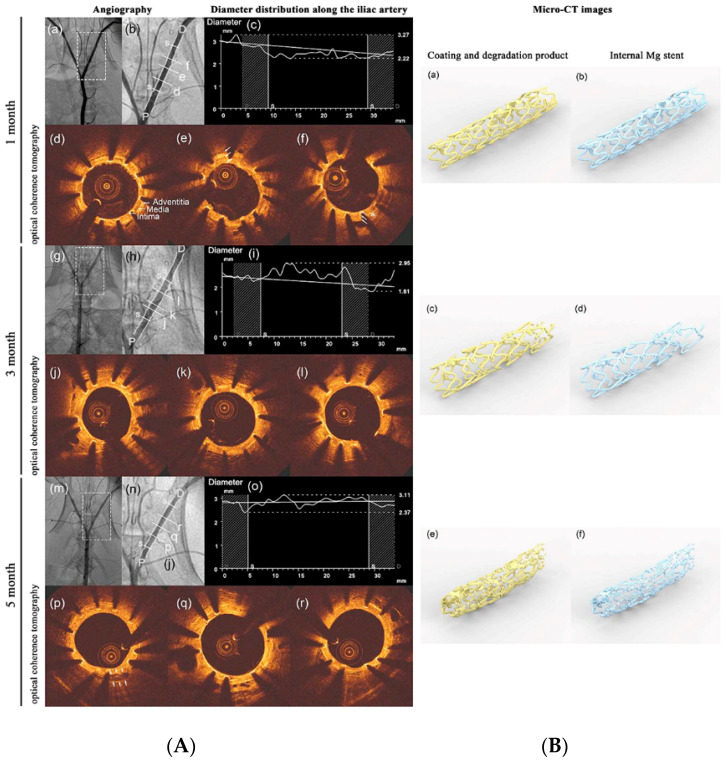
In vivo evaluation using quantitative coronary angiography (QCA), optical coherence tomography (OCT), and high-resolution µ-CT one, three, and five months post-implantation. Left side (**A**): (a,b,g,h,m,n) angiography in the rabbit, and the location of the scaffolded segment. (c,i,o) The distribution of the diameter along the iliac artery. (d-f,j-l,p-r) OCT photographs in the scaffolded segment, showing the complete endothelialization and strut embedding into the vessel wall after one month of implantation. By three months, the attenuations of signal around the edges of the struts remain sharp and the area of the lumen increased. White arrows demonstrate the bright–dark–bright three-layered appearances corresponding to intima, media, and adventitia. The asterisks show the homogeneous signal-rich regions corresponding to fibrous plaques. The double arrows indicate the degraded implant, normal arterial structures, and some calcific plaques after five months. Right side (**B**): µ-CT images. (a,b) One month after implantation, degradation was insignificant. (c,d) By three months, minimal volume loss could be seen. (e,f) At five months, OPT stent considerably degraded. Reprinted with permission from Ref. [187]. Copyright 2019, Elsevier.

Surface modification is a major approach to decelerate the degradation of Mg alloys for cardiovascular applications [101,183]. A shining example is AZ31 coronary stents laser-cut, acid pickled, and dip-coated in the solution of PCL with 1% TiO_2_. In this research, the degradation rate of AZ31 uncoated control stents was higher than AZ31 coated stents. While uncoated stents in flowing Hank’s solution lost ∼27% in weight, coated stents lost only ∼9% in weight after four weeks of dynamic degradation [184]. For cardiovascular applications, drug-eluting coatings might reduce the incidence of restenosis and optimize the corrosion profiles of Mg substrate. Tang et al. [185] applied paclitaxel incorporated in poly (trimethylene carbonate) on the surface of Mg. This coating, which was uniform, gradually degraded from surface to inside and provided long-term protection; as a result, it could be a good candidate as a drug-eluting coating for Mg-based stents. In another research, an asymmetric coating consisting of an inner PEI single layer and an outer sirolimus-loaded PLGA/PEI double layer was developed on the surface of the WE43 Mg-alloy stent. It was shown that the PEI coating layer had desirable adhesiveness to the surface of the substrate and significantly enhanced in vitro endothelial cell compatibility and the corrosion resistance of the Mg alloy, whereas the PLGA/PEI double-coating layer ensured a stable surface morphology and a low release rate of sirolimus during the drug-release process; therefore, this system could have the potential to suppress in-stent restenosis and improve re-endothelialization in vascular stent applications [186]. Chen et al. [187] applied a rapamycin-eluting polymer coating on the surface of biodegradable Mg–Nd–Zn–Zr alloy stents. An in vivo test of the optimized coated stents was performed in the iliac artery of New Zealand white rabbit with quantitative coronary angiography, optical coherence tomography, and micro-CT observation at one-, three-, and five-month follow-ups (Figure 2). According to angiography exams, neither early in-scaffold restenosis nor thrombus was observed, and the coated stents allowed for arterial healing and supported the vessel effectively before degradation. Regarding optical coherence tomography, strut embedding into the vessel wall and endothelialization occurred at one-month post-implantation. The following optical coherence tomography observation indicated that the attenuations of signal around the edges of the struts remained sharp and the lumen area increased by three months. As can be seen in micro-computed tomography scanning of the entire scaffolded-segments vessels, the degradation process of the coated stent was insignificant at one month, whereas, after five months the mechanical integrity was lost and the stent degraded significantly. Finally, these results revealed that the degradation of this stent was layer by layer from the outside to the inside.

Generally, an ideal stent needs to fulfill not only anti-restenosis and fast endothelialization but also anti-inflammation and suitable durability. By way of illustration, Ye et al. [188] fabricated a multifunctional stent by using atorvastatin calcium (ATVC) loaded into the surface-eroding poly (1,3-trimethylene carbonate) (PTMC) on the surface of AZ31 wire to obtain vascular remodeling, target drug delivery, and well-controllable degradation performance. They indicated that the degradation rate of the coated Mg was reduced in the microfluidic-chip, electrochemical, in vitro, and in vivo tests. The in vivo rat test showed that the PTMC–ATVC coating reduced intimal hyperplasia and inflammation and regulated endothelial and smooth muscle cells. Moreover, the target atorvastatin delivery demonstrated a promising dual-function coating for enhancing the early endothelialization and the durability of these stents.

**Figure 3:**
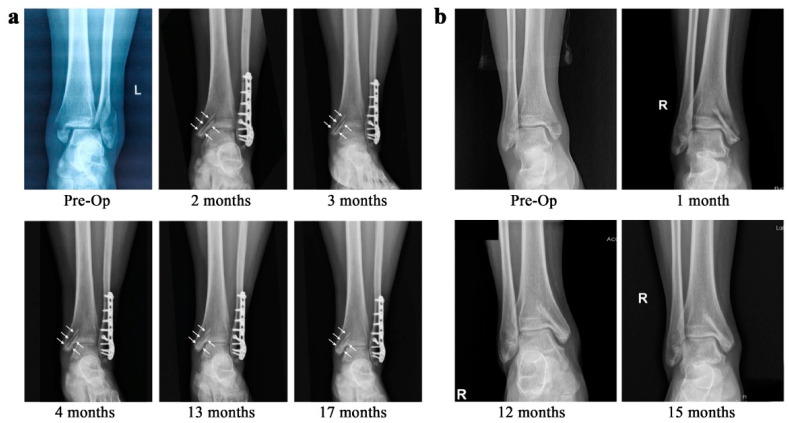
(**a**) Preoperative and postoperative radiographs of a young female patient with a trimalleolar fracture. Two Mg–Nd–Zn–Zr alloy screws coated by Ca–P coating (white arrows) were implanted for the treatment of the medial malleolar fracture. Both screws did not indicate signs of failure before fracture healing as they maintained their morphology. The radiographs also indicated the degradation process seventeen months post-surgery. (**b**) Preoperative and postoperative radiographs of a mid-age female patient with a medial malleolar fracture. The patient’s radiograph indicated radiolucent zones around screws one month postoperatively, which almost disappeared twelve months postoperatively. L and R show Left medial malleolus and Right medial malleolus. Reprinted with permission from Ref. [197]. Copyright 2021, Elsevier.

Having the ability to promote in vivo bone healing and regeneration and the mechanical properties similar to that of bones, Mg alloys with suitable coatings have the potential for use as biodegradable orthopedic implants [189,190,191]. These materials coated with calcium phosphate coatings based on hydroxyapatite and its various chemical analogues can further enhance biocompatibility [192], bioactivity [193], wear resistance [194], bone conduction, bone induction, and the degradation resistance of Mg biomaterials [195]. Gao et al. [196] deposited calcium phosphate coating containing dicalcium phosphate dihydrate on an AZ60 alloy via the chemical conversion technique. The in vitro and in vivo results indicated that this coating significantly improved the biocompatibility and biodegradation behavior of the Mg alloy. To provide a solid basis for further clinical translation, the safety and effectiveness of Mg–Nd–Zn–Zr alloy screws coated by Ca–P coating for the treatment of medial malleolar fractures was evaluated [197]. In this study, these modified Mg screws were used to treat nine patients with medial malleolar fractures (Figure 3). Postoperative radiography showed that obvious degradation occurred twelve months postoperatively and all patients achieved good medial malleolar fracture alignment. No one experienced malunion, failure of internal fixation, infection, or breakage of the screws before fracture healing. These results confirm that Ca–P-coated Mg–Nd–Zn–Zr alloy has excellent prospects for clinical translation and can be an alternative internal fixation device for fracture treatment. In a study, Husak et al. [198] applied hydroxyapatite coatings on the surface of Mg alloy with the contents of Mg (96.25 wt.%), Al (1.85 wt.%), Nb (1.25 wt.%), and Zr (0.65 wt.%). The in vitro and in vivo results indicated that the number of adherent cells on the surface of uncoated Mg alloy was significantly less than that on the surface of hydroxyapatite-coated samples, and the degradation rate of this Mg alloy was decreased by hydroxyapatite coating. It is reported that the efficiency of hydroxyapatite-coated Mg alloys can be further improved by using a kind of antimicrobial agent, along with hydroxyapatite [199].

Other ceramic coatings could effectively suppress the rapid degradation of magnesium alloys. Lin et al. [200] used the Ti and O dual-plasma ion immersion implantation (PIII) method to fabricate a multifunctional TiO_2_ based nano-layer on ZK60 Mg alloy to improve the antimicrobial activity, osteoconductivity, and corrosion resistance of the Mg alloy. The in vitro study indicated that this TiO_2_/MgO nano-layer could control the degradation rate of Mg alloy, and the in vivo assay showed that at eight weeks post-surgery, 94% of the implant volume was still maintained, thus proving that this nano-layer not only could regulate its implant-to-bone integration effectively but also could control the degradation of Mg alloy. To stimulate bone formation and enhance osteogenic activity, osteocompatibility, and corrosion resistance of Mg-based implants, Xiong et al. [201] introduced a novel coating on the surface of Mg–1Ca. They employed bioactive Ca, Sr/P-containing silk fibroin layers on the surface of the Mg alloy.

## 5. Conclusions and Future Aspect

The biodegradability and biocompatibility of Mg-based materials make them suitable for biomedical applications. Most of the currently researched Mg-based implants, however, degraded sooner than we expected. Accordingly, is it true to mention that Mg is not the best choice as a biodegradable biomaterial and that we should possibly focus on another biodegradable metal? The major drawback in this field is the lack of accurate data. As it is well-known, numerous factors have an influence on the corrosion rate and, therefore, the degradation of magnesium. Some of these factors relate to the environment in which the corrosion resistance would be performed; as a result, it is first and foremost to mimic the real body environment for observations and measurements. The absence of organic components in most simulated body solutions used for corrosion and degradation testing but has a dramatic effect on the degradation of this metal is a case in point. It is, on the other hand, believed that designing suitable composition and surface modification can significantly control the degradation process. Concerning controlling the degradation rate, numerous Mg alloys and techniques for surface modifications have been introduced for different applications, making the field of biodegradable Mg biomaterials significantly advanced. While a great deal of research ought to show the in vivo and clinical efficacy of these modified Mg alloy biomaterials, the world is still waiting for the introduction of new methods that can control the degradation of Mg-based biomaterials and offer novel functions at the same time.

## Figures and Tables

**Table 1:** Summary of the effect of most common alloying elements on the degradation behavior of Mg alloys.

Mineral	Effect on Degradation Behavior	References
Ca	Ca concentration in magnesium alloys should be less than ~1 wt.%; excessive addition of calcium in pure magnesium deteriorating corrosion resistance.	[115,116]
Zn	Improving corrosion resistance of Mg alloys mostly at a content below ~5 wt.%.	[117,118,119]
Mn	Improving corrosion resistance by decreasing impurities with a small quantity (less than ~1 wt.%) of Mn addition.	[120]
Sr	The effect on corrosion resistance; optimum content below ~2 wt.%.	[121]
Li	Improving corrosion resistance at a concentration less than ~9 wt.% in pure Mg; reducing corrosion resistance with higher Li addition.	[122]
Zr	Zr content below ~2 wt.% improving the corrosion resistance.	[123]
REEs	Generally enhancing the corrosion resistance of Mg alloys. The corrosion resistance of Mg–light REE alloys was normally better compared to Mg–heavy REE alloys.	[124,125,126]
Al	With increasing Al-content (the maximum is reached at solubility limit of 12.7 wt.% Al), the corrosion rate of homogeneous α-phase decreases.	[127]
